# Incidence Rates and Risk Factors of *Clostridioides difficile* Infection in Solid Organ and Hematopoietic Stem Cell Transplant Recipients

**DOI:** 10.1093/ofid/ofz086

**Published:** 2019-02-19

**Authors:** Emma E Ilett, Marie Helleberg, Joanne Reekie, Daniel D Murray, Signe M Wulff, Mark P Khurana, Amanda Mocroft, Gedske Daugaard, Michael Perch, Allan Rasmussen, Søren S Sørensen, Finn Gustafsson, Niels Frimodt-Møller, Henrik Sengeløv, Jens Lundgren

**Affiliations:** 1 PERSIMUNE Centre of Excellence, Rigshospitalet, Copenhagen, Denmark; 2 Centre for Clinical Research, Epidemiology, Modelling and Evaluation (CREME), Institute for Global Health, University College London, London, UK; 3 Department of Oncology, Rigshospitalet, Copenhagen, Denmark; 4 Department of Cardiology, Rigshospitalet, Copenhagen, Denmark; 5 Department of Surgical Gastroenterology, Rigshospitalet, Copenhagen, Denmark; 6 Department of Nephrology, Rigshospitalet, Copenhagen, Denmark; 7 Department of Clinical Medicine, University of Copenhagen, Copenhagen, Denmark; 8 Department of Microbiology, Rigshospitalet, Copenhagen, Denmark; 9 Department of Haematology, Rigshospitalet, Copenhagen, Denmark

**Keywords:** CDI, *Clostridioides difficile* infection, hematopoietic stem cell transplantation, solid organ transplantation

## Abstract

**Background:**

Transplant recipients are an immunologically vulnerable patient group and are at elevated risk of *Clostridioides difficile* infection (CDI) compared with other hospitalized populations. However, risk factors for CDI post-transplant are not fully understood.

**Methods:**

Adults undergoing solid organ (SOT) and hematopoietic stem cell transplant (HSCT) from January 2010 to February 2017 at Rigshospitalet, University of Copenhagen, Denmark, were retrospectively included. Using nationwide data capture of all CDI cases, the incidence and risk factors of CDI were assessed.

**Results:**

A total of 1687 patients underwent SOT or HSCT (1114 and 573, respectively), with a median follow-up time (interquartile range) of 1.95 (0.52–4.11) years. CDI was diagnosed in 15% (164) and 20% (114) of the SOT and HSCT recipients, respectively. CDI rates were highest in the 30 days post-transplant for both SOT and HSCT (adjusted incidence rate ratio [aIRR], 6.64; 95% confidence interval [CI], 4.37–10.10; and aIRR, 2.85; 95% CI, 1.83–4.43, respectively, compared with 31–180 days). For SOT recipients, pretransplant CDI and liver and lung transplant were associated with a higher risk of CDI in the first 30 days post-transplant, whereas age and liver transplant were risk factors in the later period. Among HSCT recipients, myeloablative conditioning and a higher Charlson Comorbidity Index were associated with a higher risk of CDI in the early period but not in the late period.

**Conclusions:**

Using nationwide data, we show a high incidence of CDI among transplant recipients. Importantly, we also find that risk factors can vary relative to time post-transplant.

*Clostridioides difficile* infection (CDI) is a leading cause of health care–associated diarrhea [1, 2]. Compared with other hospitalized patients, both solid organ (SOT) and hematopoietic stem cell transplant (HSCT) recipients have a 5-fold higher risk of CDI [3, 4], with up to 10%–33% developing CDI post-transplant [5–9]. Subsequently, those experiencing post-transplant CDI are at an increased risk of adverse events such as mortality, graft failure, and graft-vs-host disease [9–13].

Currently, it is not clear why transplantation induces such high CDI rates. Previous studies have found that most cases of CDI occur within the first month post-transplant [9, 12, 14–17]. However, risk factors established in other populations, such as antibiotic use, length of hospital stay, advanced age, and the use of proton pump inhibitors [18], have not been consistently identified in SOT or HSCT studies. As transplant recipients represent one of the most immunologically vulnerable patient populations, traditional risk factors may not apply to this group. Alternatively, these risk factors may be so prevalent in SOT and HSCT recipients that they cannot serve as independent predictors for CDI.

Most studies in SOT/HSCT populations trying to identify risk factors for CDI have used patient records from the transplant hospital, without access to patient information or follow-up after discharge. This could lead to an underestimation of CDI post-transplant, due to patients contracting CDI outside of their transplant hospital. Additionally, important pretransplant risk factors, such as previous CDI episodes or antibiotic use at nontransplant hospitals, may not have been captured.

Therefore, we aimed to determine the incidence of CDI in a large retrospective cohort of both SOT and HSCT recipients in Denmark and to investigate/identify associated risk factors. Our setting allows complete follow-up and national capture of both inpatient and outpatient CDI cases [19].

## METHODS

### Patient Population and Study Design

This was a cohort study of adults (aged ≥18 years), resident in Denmark, undergoing SOT or HSCT between January 1, 2010, and February 21, 2017, at Rigshospitalet, University of Copenhagen, Denmark. The study was approved by the Danish Data Protection Agency (2012-58-0004; RH-2016–47; 04433) and the Danish National Board of Health (3-3013-1060/1/).

### Data Sources

Data were retrieved from the Centre of Excellence for Personalised Medicine for Infectious Complications in Immune Deficiency (PERSIMUNE) [20] data warehouse, which contains demographic, clinical, and para-clinical data from electronic health records through regional and national electronic data repositories in Denmark. Microbiology data were retrieved from the Danish Microbiology Database (MiBa) and used for national surveillance of both hospital-acquired and community-acquired CDI [19]. Medication data were manually collected first from electronic medication systems and then crossed-referenced with electronic patient charts by 2 independent reviewers. For additional information regarding data sources, please see the Supplementary Data.

### *Clostridioides difficile* Testing

Due to the national nature of the microbiology data, testing for *C. difficile* could, in principle, have been performed at any of the microbiology departments in Denmark. Testing for *C. difficile* was not performed routinely and was conducted at the discretion of hospital physicians or general practitioners in Denmark during the study period. Therefore, all testing was considered to be based on clinical symptoms (ie, diarrhea).

### Definitions

CDI was defined as a positive toxigenic culture (ie, a culture with growth of *C. difficile* and which tested positive for *C. difficile* toxin genes) or a positive toxin test (ie, a positive polymerase chain reaction test for *C. difficile* toxin genes without a culture of *C. difficile*). Cultures with growth of *C. difficile* and a negative toxin test within 30 days of the original culture were not considered CDI cases. Cultures with a growth of *C. difficile* and no performed toxin test (ie, it was unknown whether the *C. difficile* bacterium found was toxigenic) were considered CDI cases. The first CDI episode after transplantation had to be >30 days from a pretransplantation CDI episode.

### Statistical Analysis

The primary outcome was the first CDI episode after transplantation. Patients were followed from transplantation until February 21, 2017, death, emigration, retransplantation, or post-transplant CDI, whichever came first.

Patient characteristics were compared for those with and without posttransplantation CDI using the Wilcoxon-Mann-Whitney (for continuous variables) and chi-square (for categorical variables) tests. If there were <5 patients in a categorical group, the Fisher exact test was used instead of the chi-square test.

To investigate whether the rates of CDI post-transplantation were elevated compared with pretransplantation rates, incidence rates of CDI were calculated including the 6 months before transplant. For this analysis, baseline was thus redefined as 180 days before the transplantation date or January 1, 2010, whichever occurred later. The crude incidence rate (IR) of CDI was calculated per 100 person-years of follow-up for each transplantation type in the following time periods: pretransplantation (up to 180 days before transplantation), early (0–30 days post-transplantation), middle (31–180 days post-transplantation), and late (>180 days post-transplantation). Crude IRs were calculated with censoring after the first CDI episode (irrelative of time period) and compared with crude IRs when repeated events were allowed (excluding 30 days between events).

Poisson regression was used to investigate the following risk factors for post-transplant CDI: pretransplantation CDI, transplant type, time from transplantation, the Charlson Comorbidity Index (CCI) [21, 22], donor/recipient serostatus for cytomegalovirus, and baseline levels of albumin, neutrocytes, and lymphocytes. The cutoffs for baseline measurements were those registered up to 14 days before transplantation to minimize measurements not reflecting the recipients’ status at the time of transplant. CCI was calculated based on all inpatient and outpatient diagnoses up to the date of transplantation. Risk factors found to be significant (*P *< .1) in univariable models were included in the multivariable model, with those that were significant (*P *< .1) after adjustment retained in the final model. Models were developed separately for SOT and HSCT recipients.

Sensitivity analyses were performed to assess whether censoring patients 1 year after transplantation and excluding those with <6 months of follow-up before transplantation altered the results. Further, the main analyses were repeated, treating death, emigration, and retransplantation as competing risks. Due to the proportionality assumption, this was done in 2 separate time periods (early [0–30 days] and combined middle and late periods [>30 days] post-transplantation).

### Nested Case–Control Study

To assess the possible effect of medication on CDI risk, we conducted a nested case–control study, matching cases and controls 1-to-1 based on transplantation type and time of transplantation. Cases were patients who contracted CDI within the first 60 days post-transplantation. Controls were patients who did not contract CDI within the first 70 days post-transplantation and had the same or a longer follow-up time relative to transplantation as their matched case. Medication data assessed included proton pump inhibitors (PPIs), laxatives, antibiotics, steroids, antimycotics, and parenteral nutrition.

Medication data were collected from 90 days preceding the CDI episode (including before transplant if necessary) for cases and the corresponding time period relative to transplantation for controls. The number of days on each type of medication was calculated as the total number of time period days (ie, when 2 different antibiotics were given on the same day, the time period days = 1).

Logistic regression analysis was used to investigate the association between medication use and post-transplant CDI, with those found to be significant (*P *< .1) in univariable analyses included in the multivariable model. Again, models were developed separately for SOT and HSCT recipients. Time period days were compared by taking the median number of days for all cases and controls including those not receiving treatment. A sensitivity analysis was performed using a cumulative number of medication days (ie, when 2 different antibiotics were given on the same day, the medication days = 2).

For additional information on medication data collection (including standard immunosuppression regimes), see the Supplementary Data and Supplementary Figure 1. For standard antibacterial, antifungal, and antiviral prophylactic regimes, please see Supplementary Table 1.

All statistical analyses were performed using SAS Enterprise Guide (version 7.1) and SAS (version 9.4; SAS Institute, Cary, NC).

## RESULTS

### Study Population

During the study period, 1687 patients underwent SOT or HSCT (1114 and 573, respectively), with a median follow-up time (interquartile range [IQR]) of 1.9 (0.5–4.0) years post-transplantation. The most common SOT was kidney (n = 548); followed by liver (n = 273), lung (n = 202), heart (n = 81), and pancreas (n = 10). Among these, 10 pancreas, 6 liver, and 1 lung transplant were combined with a simultaneous kidney transplantation. All HSCTs were allogeneic, with nonmyeloablative (n = 326) being the most common conditioning type, followed by myeloablative (n = 247). Additional patient characteristics can be found in Table 1.

**Table 1.** Characteristics for SOT and HSCT Recipients

	SOT			HSCT		
	Non-CDI	CDI	*P* Value^a^	Non-CDI	CDI	*P* Value^a^
No.	950	164		459	114	
Total person-years of follow-up	2887	132		1056	84	
Median person-years of follow-up (IQR)	2.74 (1.3–4.9)	0.10 (0.0–0.8)		1.6 (0.5–3.7)	0.3 (0.1–0.7)	
Median year of transplant (IQR)	2013 (2011–2015)	2013 (2011–2014)	.38	2013 (2011–2015)	2013 (2012–2015)	.51
Median age (IQR), y	50 (41–59)	52 (44–60)	.11	55 (43–64)	51 (38–61)	.04
Age group, No. (%)						
<40	214 (23)	30 (18)	.39	97 (21)	31 (27)	.07
40–49	250 (26)	39 (24)		89 (19)	24 (21)	
50–59	284 (30)	53 (32)		90 (20)	28 (25)	
≥60	202 (21)	42 (26)		183 (40)	31 (27)	
Male gender, No. (%)	583 (61)	96 (59)	.5	276 (60)	69 (61)	.93
Donor-recipient CMV serostatus,^b^ No. (%)						
High	169 (18)	23 (14)	.64	144 (31)	31 (27)	.747
Intermediate	373 (39)	66 (40)		153 (33)	40 (35)	
Low	325 (34)	61 (37)		124 (27)	35 (30)	
Missing	83 (9)	14 (9)		38 (8)	8 (7)	
Median Charlson Comorbidity Index (IQR)	3 (2–4)	3 (2–4)	.05	2 (0–2)	2 (1–3)	.29
Baseline^c^ lymphocytes, No. (%)						
Below normal (<1.0*10^9^/L)	263 (28)	64 (39)	.001	210 (46)	52 (46)	.51
Normal (1.0–<3.5*10^9^/L)	598 (63)	77 (47)		226 (49)	58 (51)	
Above normal (≥3.5*10^9^/L)	14 (2)	2 (1)		18 (4)	4 (4)	
Missing	75 (8)	21 (13)		5 (1)	0 (0)	
Baseline^c^ neutrocytes, No. (%)						
Below normal (<1.6*10^9^/L)	12 (1)	4 (2)	.10	164 (36)	33 (29)	.11
Normal (1.6–<5.9*10^9^/L)	500 (53)	81 (50)		254 (55)	74 (65)	
Above normal (≥5.9*10^9^/L)	365 (38)	58 (35)		34 (7)	7 (6)	
Missing	73 (8)	21 (13)		7 (2)	0 (0)	
Baseline^c^ albumin,^d^ No. (%)						
Below normal	307 (32)	77 (47)	.005	16 (4)	4 (4)	.99
Normal	375 (39)	49 (30)		200 (44)	48 (42)	
Above normal	77 (8)	10 (6)		55 (12)	14 (12)	
Missing	191 (20)	28 (17)		189 (41)	48 (42)	
Pretransplantation CDI, No. (%)						
Never	919 (97)	150 (92)	.004	422 (92)	102 (90)	.1
Yes (not in 6 mo before Tx)	27 (3)	9 (6)		13 (3)	1 (1)	
Yes (in 6 mo before Tx)	4 (0.4)	5 (3)		24 (5)	11 (10)	

Abbreviations: CDI, *Clostridioides difficile* infection; CMV, cytomegalovirus; HSCT, hematopoietic stem cell transplant; IQR, interquartile range; SOT, solid organ transplant.

^a^ *P* values were determined using the Wilcoxon-Mann-Whitney test for continuous variables and the chi-square test for categorical variables (with missing values as a category, so that all patients were included). If there were <5 patients in a categorical group, the Fisher exact test was used instead of the chi-square test.

^b^High for SOT = donor (D)+/recipient (R)-; high for HSCT = D-/R+; intermediate for SOT and HSCT = D+/R+; low for SOT = D-/R+; low for HSCT = D+/R-.

^c^Baseline measurements were those taken up to 14 days before transplantation.

^d^Levels for albumin: normal: age 18–39: 36–48 g/L; age 40–69: 36–45 g/L; age 70–125: 34–45 g/L; below: levels under normal per age group; above: levels above normal per age group.

### CDI Cases

A total of 164 (15%) SOT recipients and 114 (20%) HSCT recipients developed CDI post-transplant. Of these cases, 73 (45%) and 63 (55%) were based on a positive toxin test, 65 (40%) and 41 (36%) on a toxigenic culture, and 26 (16%) and 10 (9%) on a positive culture for *C. difficile* without toxin testing for SOT and HSCT cases, respectively. Additional information on CDI cases and testing methods over time can be found in the Supplementary Data.

The majority of CDI cases were hospital inpatients at the time of diagnosis (85% [n = 142] and 71% [n = 80] of SOT and HSCT cases respectively). Among the outpatient cases, only 46% of SOT recipients were tested for *C. difficile* at their transplant hospital (ie, Rigshospitalet), whereas 75% of HSCT outpatient cases were tested via the outpatient clinic at Rigshospitalet. When combining in- and outpatient cases of CDI, *C. difficile* testing had been ordered by the transplanting hospital (Rigshospitalet) in most cases; however, 27% of SOT cases and 15% of HSCT cases had had their *C. difficile* test ordered at an alternative hospital or health care facility.

### CDI Incidence Rates

**Figure 1.**
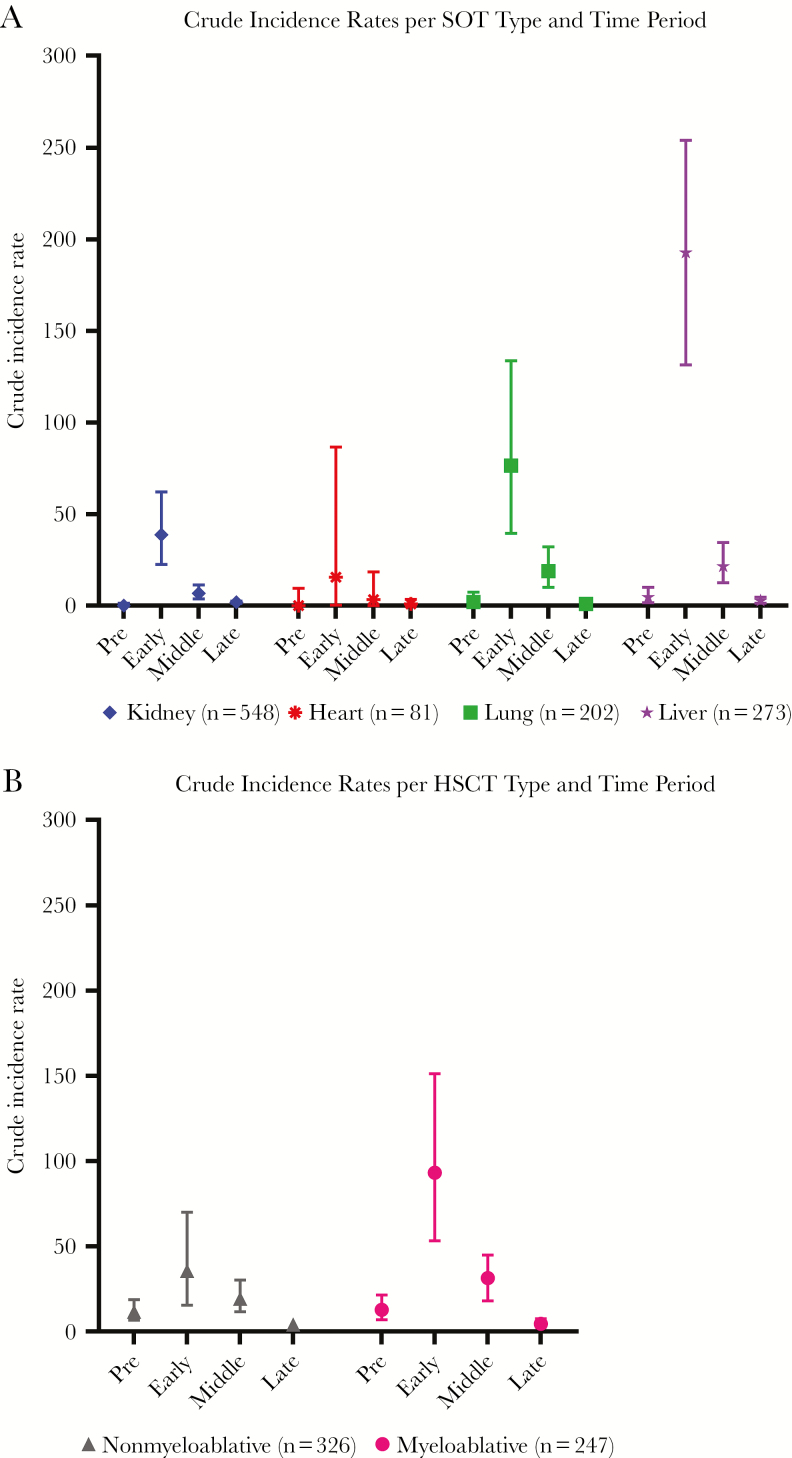
A, Crude incidence rates per 100 person-years of follow-up and 95% confidence intervals for each solid organ transplant type in the following time periods relative to transplantation: Pre: time before transplantation; Early: 0–30 days post-transplantation; Middle: 31–180 days post-transplantation; Late: >180 days post-transplantation. B, Crude incidence rates per 100 person-years of follow-up and 95% confidence intervals for each hematopoietic stem cell transplant type in the following time periods relative to transplantation: Pre: time before transplantation; Early: 0–30 days post-transplantation; Middle: 31–180 days post-transplantation; Late: >180 days post-transplantation. Abbreviations: HSCT, hematopoietic stem cell transplant; SOT, solid organ transplant.

The crude incidence rate (IR) of CDI varied considerably in the different time periods relative to transplantation (Figure 1). In SOT recipients, the incidence of CDI was low in the 6-month pretransplant period (1.5/100 PYFU; 95% confidence interval [CI], 0.7–3.0), peaking dramatically in the first month post-transplant (78.7; 95% CI, 60.0–97.4) and falling in the middle period (11.6; 95% CI, 8.2–15.0) but not reaching pretransplant levels until the late post-transplant period (1.9; 95% CI, 1.3–2.4). Among the HSCT group, the IR in the pretransplant period was higher than in SOT recipients (11.6; 95% CI, 7.5–15.7). However, the highest rate was again in the first month post-transplant (61.2; 95% CI, 37.6–84.7), decreasing to the lowest rate in the late post-transplant period (4.2; 95% CI, 2.8–5.6). This trend was consistent when repeated events were included.

### Risk Factors for Post-transplant CDI

Poisson regression analysis found that transplant type, age, CDI before transplantation, and time since transplantation were all significantly associated with increased risk of CDI post-SOT (Table 2; Supplementary Table 2). After adjusting for these factors, the first 30 days post-transplant were associated with a 6.64 times increased risk of CDI (95% CI, 4.37–10.10) compared with the middle post-transplant period. Additionally, liver recipients had a higher risk of CDI compared with kidney recipients (adjusted IR ratio [aIRR], 3.17; 95% CI, 2.23–4.49), and pretransplant CDI was significantly associated with an increased risk of post-transplant CDI.

**Table 2.** Risk Factors for CDI by Post-transplantation Time Period

SOT^a^						
	All Time Periods Post-transplant^b^		Early Time Period		Middle/Late Time Period	
	Multivariate IRR (95% CI)	*P* Value	Multivariate IRR (95% CI)	*P* Value	Multivariate IRR (95% CI)	*P* Value
Transplant type						
Kidney	1.00		1.00		1.00	
Heart	0.50 (0.18–1.36)	.1713	0.39 (0.05–2.94)	.3605	0.55 (0.17–1.78)	.315
Lung	1.52 (0.96–2.40)	.0738	3.03 (1.62–5.67)	.0005	0.86 (0.41–1.80)	.6829
Liver	3.17 (2.23–4.49)	<.0001	5.82 (3.39–9.97)	<.0001	1.84 (1.08–3.14)	.0243
Age at transplant (per 10 y older)	1.17 (1.02–1.34)	.0278	1.08 (0.90–1.28)	.4134	1.34 (1.07–3.14)	.0111
Pretransplant CDI^c^						
No	1.00		1.00		1.00	
Yes (≥6 mo before Tx)	2.43 (1.17–5.03)	.0172	4.35* (2.23–8.47)	<.0001	1.09* (0.25–4.83)	.9095
Yes (in 6 mo before Tx	5.67 (1.89–17.02)	.002				
HSCT						
	All Time Periods Post-transplant^b^		Early Time Period		Middle/Late Time Period	
	Multivariate IRR (95% CI)	*P* Value	Multivariate IRR (95% CI)	*P* Value	Multivariate IRR (95% CI)	*P* Value
Transplant type						
Nonmyeloablative	1.00		1.00		1.00	
Myeloablative	1.72 (1.17–2.25)	.0057	2.88 (2.53–5.42)	.0011	1.20 (0.72–1.99)	.4851
Year of transplantation (grouped)						
2010–2011	1.00		1.00		1.00	
2012–2013	2.25 (1.30–3.89)	.0036	3.58 (1.18–10.81)	.0239	2.12 (1.11–4.08)	.0234
2014–2015	3.23 (1.87–5.60)	<.0001	5.91 (2.06–16.95)	.001	3.33 (1.68–6.59)	.0006
2016–2017	1.70 (0.77–3.75)	.1887	2.62 (0.73–9.34)	.1387	3.41 (1.12–10.41)	.0311
CCI score						
Per point higher	1.17 (1.02–1.33)	.0224	1.20 (1.00–1.44)	.0453	1.17 (0.95–1.44)	.1434

Abbreviations: CCI, Charlson Comorbidity Index; CDI, *Clostridioides difficile* infection; CI, confidence interval; HSCT, hematopoietic stem cell transplant; OR, odds ratio; SOT, solid organ transplant.

^a^Due to no CDI cases, pancreas recipients were removed from the analyses.

^b^Also adjusted for time since transplantation.

^c^Pretransplant CDI <6 months before transplant or ≥6 months before transplant were combined when looking at early and middle/late time periods separately.

Factors associated with post-transplant CDI in HSCT recipients were conditioning type, year of transplant, time since transplantation, and CCI (Table 2; Supplementary Table 2). As with SOT, the greatest risk of CDI in HSCT recipients was within the first month post-transplant (aIRR, 2.85; 95% CI, 1.83–4.43) compared with the middle post-transplant period). Further, myeloablative recipients were associated with a higher risk of post-transplant CDI than nonmyeloablative recipients (aIRR, 1.72; 95% CI, 1.17–2.25), and a higher CCI score was also associated with an increased risk (aIRR, 1.17; 95% CI, 1.02–1.33 per point higher).

### Changes in Risk Factors Based on Time From Transplantation

There was a significant interaction between time since transplantation and transplant/conditioning type within the SOT (*P* = .02) and HSCT groups (*P* < .0001). To further assess risk factors relative to time after transplantation, the analysis was split into 2 time periods: early (≤30 days post-transplant) and middle/late (>30 days post-transplant) (Table 2). Among SOT recipients, lung recipients were found to have an increased risk of CDI in the early post-transplant period compared with kidney recipients (aIRR, 3.03; 95% CI, 1.62–5.67). Also, pretransplant CDI was only significant in the early time period, whereas age was only a significant risk factor in the middle/late time period. Among HSCT recipients, transplantation type and CCI were the only significant risk factors for CDI in the early post-transplant period.

### Sensitivity Analyses and Death as a Competing Risk

Results of the sensitivity analyses censoring patients 1 year after transplantation and excluding those with <6 months follow-up before transplant were consistent with the main analyses. Competing risk analyses were similar to the main analyses for HSCT. In the SOT group, the hazard ratio for lung recipients compared with kidney in the early post-transplant period became nonsignificant (hazard ratio, 1.95; 95% CI, 0.93–4.08). However, only 3 lung recipients died within the early post-transplant period.

### Case–Control Study for Medication Use

A total of 216 patients were included in the case–control study looking at the association between exposure to medication groups and risk of CDI (SOT n = 132, HSCT n = 84). The number of patients prescribed each medication and the median number of treatment days among those who were prescribed the medication are shown in Table 3. There was no significant association between the total number of time period days with antibiotics, antimycotics, steroid treatment, or parenteral nutrition and CDI in either the SOT group or HSCT group.

**Table 3.** Nested Case–Control Study of Medication Use 90 Days Before CDI

	SOT^a^			HSCT		
	Cases (n = 66)	Controls (n = 66)	*P* Value	Case (n = 42)	Control (n = 42)	*P* Value
Age, median (IQR), y	50 (42–57)	54 (44–60)	.16	48 (40–60)	52 (39–62)	.53
Male, No. (%)	41 (62)	36 (55)	.47	24 (57)	22 (52)	.66
Charlson Comorbidity Index, median (IQR)	3 (2–4)	3 (1–4)	.99	2 (2–3)	2 (2–3)	.42
Antibiotics^b^						
Received treatment, No. (%)	66 (100)	66 (100)	—	42 (100)	42 (100)	—
Median^c^ No. of time period days (IQR)	30 (11–48)	22 (6–68)	.84	59 (43–80)	52 (34–65)	.09
Antibiotic groups^b,d^						
Clindamycin						
Received treatment, No. (%)	1 (2)	0	1.00	1 (2)	4 (10)	.35
Median^c^ No. of time period days (IQR)	16 (–)	—	—	9 (–)	13 (7–23)	.71
Fluoroquinolones						
Received treatment, No. (%)	39 (59)	33 (50)	.29	37 (88)	37 (88)	1.00
Median^c^ No. of time period days (IQR)	12 (6–29)	15 (6–26)	.89	29 (14–44)	22 (13–38)	.19
3rd/4th-generation cephalosporins						
Received treatment, No. (%)	27 (41)	26 (39)	.85	29 (69)	18 (43)	.01
Median^c^ No. of time period days (IQR)	6 (4–7)	5 (5–6)	.41	8 (5–11)	7.5 (4–11)	.80
Piperacillin/tazobactam						
Received treatment, No. (%)	8 (12)	0	.006	15 (36)	16 (38)	.82
Median^c^ No. of time period days (IQR)	7 (5–43)	—	—	2 (1–5)	4 (3–14)	.03
Carbapenems						
Received treatment, No. (%)	40 (61)	27 (41)	.02	31 (74)	28 (67)	.47
Median^c^ No. of time period days (IQR)	13 (5–20)	11 (6–23)	.75	13 (7–20)	12 (6–19)	.78
Beta-lactam/beta-lactamase inhibitor comb. (excl. piperacillin/tazobactam)						
Received treatment, No. (%)	37 (56)	33 (50)	.48	30 (71)	31 (74)	.80
Median^c^ No. of time period days (IQR)	6 (4–7)	5 (4–6)	.31	15 (7–26)	14 (7–19)	.64
Other antibiotics						
Received treatment, No. (%)	60 (91)	58 (88)	.77	42 (100)	42 (100)	—
Median^c^ No. of time period days (IQR)	20 (7–35)	22 (5–52)	.58	31 (19–60)	22 (10–39)	0.05
No. of different antibiotic medications prescribed^e^ (IQR)	3 (2–4)	3 (2–3)	.02	5 (3–5)	4.5 (3–5)	.39
1–2, No. (%)	19 (29)	26 (39)		4 (10)	3 (7)	
3–4, No. (%)	35 (53)	37 (56)		14 (33)	18 (43)	
>5, No. (%)	12 (18)	3 (5)		24 (57)	21 (50)	
Proton pump inhibitors^b^						
Received treatment, No. (%)	66 (100)	65 (99)	1.00	36 (86)	32 (76)	.26
Median^c^ No. of time period days (IQR)	49 (15–90)	28 (8–79)	.03	45 (29–81)	33 (21–64)	.15
Steroids^b^						
Received treatment, No. (%)	66 (100)	66 (100)	—	24 (57)	17 (41)	.12
Median^c^ No. of time period days (IQR)	21 (12–46)	19 (9–36)	.17	9 (4–26)	10 (6–21)	.57
Antimycotics^b^						
Received treatment, No. (%)	48 (73)	46 (70)	.70	42 (100)	41 (98)	1.00
Median^c^ No. of time period days (IQR)	12 (8–31)	10 (7–21)	.35	52 (36–69)	44 (30–63)	.32
Parenteral nutrition^b^						
Received treatment, No. (%)	1 (2)	0	1.00	23 (55)	23 (55)	1.00
Median^c^ No. of time period days (IQR)	5 (–)	—	—	12 (8–24)	12 (6–20)	.57
Received laxatives^f^	38 (58)	34 (52)	.48	15 (36)	7 (17)	.04

Abbreviations: CDI, *Clostridioides difficile* infection; HSCT, hematopoietic stem cell transplant; IQR, interquartile range; SOT, solid organ transplant.

^a^Due to no CDI cases, there were no pancreas recipients in the nested case–control study.

^b^Within 90 days before CDI for cases or corresponding time period relative to transplantation for controls.

^c^Median number of time period days only including those receiving the medication in question. Different types of medications within medication groups are not counted cumulatively; max median number of days is 90.

^d^Antibiotic subgroups are listed in the Supplementary Data.

^e^Number of different antibiotic medications prescribed within 90 days before CDI for cases or corresponding time period relative to transplantation for controls.

^f^If patients received laxatives up to 7 days before CDI for cases or corresponding time period relative to transplantation for controls.

Among the SOT group, only the number of different antibiotic groups prescribed was significantly associated with increased odds of CDI among cases compared with controls in the multivariate model after adjusting for age, sex, transplant type, year of transplant, and CCI with an adjusted odds ratio (aOR) of 1.66 (95% CI, 1.07–2.57) per extra medication group. In the univariable analysis, the total number of time period days with carbapenems was associated with higher odds of CDI (OR, 1.09; 95% CI, 1.02–1.18); however, this did not remain significant in the multivariable model (aOR, 1.04; 95% CI, 0.98–1.09). The difference in use of carbapenems between cases and controls was most prominent in liver recipients (Supplementary Table 3). Among the HSCT group, only treatment days with other antibiotics was associated with significantly higher odds of CDI in the multivariable model after adjusting for sex, age, conditioning type and year of transplant, and CCI (aOR, 1.02; 95% CI, 1.00–1.05 per extra treatment day). This difference was most prominent in the nonmyeloablative group (Supplementary Table 3). Laxative use in the week before CDI was also borderline significant in the multivariate analysis of HSCT cases compared with controls (aOR, 2.91; 95% CI, 0.94–9.00; *P *= .065).

Sensitivity analyses previously described for cumulative medication were consistent with the above results. The univariate and multivariate logistic regression analyses for the nested case–control study can be found in Supplementary Table 4.

## DISCUSSION

This study gives a comprehensive overview of the dynamics of CDI relative to transplantation, with national data capture of both inpatient and outpatient cases, as well as extensive follow-up. Consistent with other studies, we found that CDI is a common complication of transplantation and that the first month post-transplant is a high-risk period for recipients [9, 12, 14–17]. Furthermore, we identified several CDI risk factors that vary depending on time post-transplant.

A key aspect of our study was access to national microbiology data. As such, we were able to observe the incidence of CDI both pre- and post-transplant, across different hospitals and general practitioners irrespective of discharge from our transplant hospital. This nationwide data capture showed that post-transplant CDI affected 15% and 20% of SOT and HSCT recipients, respectively. Previously reported rates of CDI have generally been between 2% and 10% among SOT recipients [4, 5, 10, 11, 23] and 8% and 15% among HSCT recipients [12, 14–16, 24–29], with a small number of HSCT studies also reporting rates >20% [6, 30]. All the HSCTs in this study were allogenic. As allogenic HSCTs have a higher incidence of CDI [25], this may partly explain the higher observed incidence. However, our data also captured 29% of HSCT cases and 14% of SOT cases who were outpatients at the time of CDI diagnosis. Additionally, 15% and 27% of our HSCT and SOT cases were tested at alternative hospitals or health care facilities and not at our transplant hospital. These additional data could help explain the higher rates observed in our cohort and may better reflect the true burden of CDI post-transplant.

When assessing risk factors for post-transplant CDI, previous SOT studies have observed a greater risk associated with both liver and lung transplant recipients compared with kidney recipients [10, 11]. Our findings support this; however, in our study, lung recipients had a higher risk of CDI only when assessing the first 30 days post-transplant separately from the whole study period. Among HSCT recipients, previous studies have observed a higher risk of CDI in myeloablative recipients compared with nonmyeloablative recipients [12, 31]. This is again similar to our results; however, when assessing the early and middle/late periods separately, the increased risk found in myeloablative recipients was only observed in the first 30 days post-transplant.

These observations of variation in the risk factors of CDI are important findings and suggest that host changes relative to time since transplant may entail different responses to *C. difficile* exposure. Host changes could include recipients being at their most immunologically vulnerable time point in the early post-transplant period. This might be due to the varying degrees of injury to their own immune system (obliteration in HSCT recipients or postsurgery effects in SOT recipients) while simultaneously starting immunosuppression (as to not reject the new immune system/organ). Alternatively, it is possible that there is a testing bias in our cohort. A treating physician may be more likely to order a stool culture early after transplantation, when recipients can be more clinically vulnerable. If there is a substantial proportion of *C. difficile* colonization, as found in previous HSCT studies [29], this could lead to false-positive results, as the bacteria found in a colonized patient could be an incidental finding and not the actual cause of diarrhea.

Medication use did not appear to greatly affect risk of CDI in our study. There was a trend observed in SOT recipients of an increased risk of CDI with a greater number of different antibiotics received; however, this trend was not seen in HSCT recipients. Furthermore, it is worth noting the high prevalence medications received among all the patients in our study; for example, all patients received antibiotics, with the majority receiving at least 3 different antibiotics during the 90-day observation period. Thus, we cannot rule out the possibility that antibiotics and other medications play a major role in the development of CDI among transplant recipients but that their prevalence is so high in this population that we could not clearly identify it as an independent predictor.

Limitations to our study include not knowing possible colonization of recipients before transplantation and not typing *C. difficile* strains. Knowing the *C. difficile* strain of each CDI case could indicate if patients were infecting each other. However, cases of CDI were not isolated incidences over time (Supplementary Figure 2). Furthermore, lung and heart recipients were treated in the same ward at our hospital (Rigshospitalet), and the large difference in CDI incidence between these transplant types suggests that cross-contamination is unlikely. An additional consideration is that pretransplant rates of CDI were calculated by looking back from the start of follow-up (time of transplantation), and, although unlikely, there may have been individuals with a CDI in the period who did not continue to have a transplant or where the transplant was delayed, who were thus not included in the analysis.

In conclusion, we identify a high-risk period for CDI within the first month after transplantation in a large cohort with national data capture of all CDI cases and extensive follow-up. This high-risk period appears to be particularly prominent for liver, lung, and myeloablative HSCT recipients. In addition, we find that risk factors can vary depending on time from transplantation. These are important observations, both for designing future studies to derive risk scores for CDI in transplant recipients and to give a more comprehensive clinical picture of CDI in an immunologically vulnerable cohort.

## Supplementary Data

Supplementary materials are available at *Open Forum Infectious Diseases* online. Consisting of data provided by the authors to benefit the reader, the posted materials are not copyedited and are the sole responsibility of the authors, so questions or comments should be addressed to the corresponding author.

Supplementary_Figure_1

Supplementary_Figure_2

Supplementary_Table_1

Supplementary_Table_2

Supplementary_Table_3

Supplementary_Table_4

Supplementary_Material_1

Supplementary_Material_2

Supplementary_Material_3
