# Supplementary material for: Incidence Rates and Risk Factors of Clostridioides difficile Infection in Solid Organ and Hematopoietic Stem Cell Transplant Recipients
Source: Open Forum Infect Dis. 2019 Feb 19;6(4):ofz086. doi: 10.1093/ofid/ofz086 (PMC6441586; doi:10.1093/ofid/ofz086)
Supplement: Supplementary_Figure_2 [file ofz086_suppl_supplementary_figure_2.docx]

# **Supplementary Figure 2**

## CDI over time per transplant group
