# Supplementary material for: Incidence Rates and Risk Factors of Clostridioides difficile Infection in Solid Organ and Hematopoietic Stem Cell Transplant Recipients
Source: Open Forum Infect Dis. 2019 Feb 19;6(4):ofz086. doi: 10.1093/ofid/ofz086 (PMC6441586; doi:10.1093/ofid/ofz086)
Supplement: Supplementary_Table_1 [file ofz086_suppl_supplementary_table_1.docx]

# Supplementary Table 1

# Antibiotic, antifungal and antiviral prophylaxis for SOT and HSCT

| Transplant type | Antibiotic prophylaxis | Antifungal prophylaxis | Antiviral prophylaxis |
| --- | --- | --- | --- |
| Heart | Cefuroxime 1.5 g x3 i.v. day 0-2.  Sulfamethoxazole + trimethoprim 400/80 mg x1 p.o. day 0-lifelong. | Nystatin 5 ml x4 p.o. day 1-30. | Valganciclovir* 450 mg x2 p.o. day 0-90.  If donor and recipient are CMV seronegative: valaciclovir* 500 mg x2 p.o. day 0-90. |
| Kidney | Cefuroxime 1.5 g x1 i.v. day 0.  Mecillinam 400 mg x3 p.o. from the day before until the day after removal of the recipient’s JJ-catheter.  Sulfamethoxazole + trimethoprim 400/80 mg x1 p.o. day 0-180. |  | Valganciclovir* 450 mg x1 p.o., every other day, day 0-90. |
| Liver | Meropenem 2g x1 i.v. day -1.  Meropenem 1g x3 i.v. day 0-5. | Nystatin 1ml x4 p.o. until able to consume food. | Valganciclovir* 450 mg x2 p.o. day 0-90  If donor is CMV seronegative: valaciclovir* 500 mg x2 p.o. day 0-90. |
| Lung | Meropenem 2 g i.v. x1 day 0.  Ciprofloxacin 400 mg i.v. x1 day 0.  Meropenem 1 g i.v. x3 day 1.  Ciprofloxacin 400 mg x2 i.v. day 1.  Ciprofloxacin 500 mg p.o. x2 day 2.  Sulfamethoxazole + trimethoprim 400/80 mg x1 p.o. day 0-lifelong. | Voriconazole 200 mg x2 p.o. (can depend on blood concentrations) day 0 – 90.  Nystatin 5 ml x4 p.o. day 1 or 2 until prednisolone doses are ≤ 10 mg (approx. 14-21 days). | Valganciclovir* 450 mg x2 p.o. or ganciclovir 5 mg/kg x1 i.v. day 0-90.  If donor and recipient are CMV seronegative: valaciclovir* 500 mg x2 p.o. day 0-90. |
| Myeloablative HSCT | When the neutrophil count is <0.5 x10^9/l: ceftazidime 2 g t.i.d. until neutrophil count >0.5 x10^9/l.  Sulfamethoxazole + trimethoprim 400/80 mg x1 p.o. day 0- until end of immunosuppressive treatment. | Fluconazole 400 mg x1 i.v./p.o. day 0-75. | Acyclovir 5 mg/kg x3 p.o./i.v. day 0-180. |
| Non-myeloablative HSCT | When the neutrophil count is <0.5 x10^9/l: ciprofloxacin 500 mg x2 p.o. until neutrophil count >0.5 x10^9/l.  Sulfamethoxazole + trimethoprim 400/80 mg x1 p.o. day 0- until end of immunosuppressive treatment. | Fluconazole 400 mg x1 i.v./p.o. day 0-75. | Acyclovir 5 mg/kg x3 p.o./i.v. day 0-180. |

*For all receiving valganciclovir or valaciclovir, dosage can vary dependent on kidney function.

All medications are given daily unless otherwise stated.
