# Supplementary material for: Incidence Rates and Risk Factors of Clostridioides difficile Infection in Solid Organ and Hematopoietic Stem Cell Transplant Recipients
Source: Open Forum Infect Dis. 2019 Feb 19;6(4):ofz086. doi: 10.1093/ofid/ofz086 (PMC6441586; doi:10.1093/ofid/ofz086)
Supplement: Supplementary_Table_2 [file ofz086_suppl_supplementary_table_2.docx]

# Supplementary Table 2

# Results of the univariate and multivariate Poisson analyses

| SOT^1^ | | | | | | | | |
| --- | --- | --- | --- | --- | --- | --- | --- | --- |
|  | | | Univariate IRR  (95% CI) | P-value | Multivariate IRR  (95% CI) | P-value | Final multivariate model IRR  (95% CI) | P-value |
| Overall | | |  |  |  |  |  |  |
| Transplant type | | |  |  |  |  |  |  |
|  | Kidney | | 1.00 |  | 1.00 |  | 1.00 |  |
|  | Heart | | 0.48 (0.17-1.34) | 0.1607 | 0.41 (0.13-1.27) | 0.1207 | 0.50 (0.18-1.36) | 0.1713 |
|  | Lung | | 1.77 (1.09-2.85) | 0.0198 | 1.21 (0.64-2.31) | 0. 557 | 1.52 (0.96-2.40) | 0.0738 |
|  | Liver | | 3.62 (2.47-5.28) | <.0001 | 2.83 (1.81-4.43) | <.0001 | 3.17 (2.23-4.49) | <.0001 |
| Gender | | |  |  |  |  |  |  |
|  | Male | | 1.00 |  |  |  |  |  |
|  | Female | | 1.05 (0.75-1.46) | 0.79 |  |  |  |  |
| Age at transplant | | |  |  |  |  |  |  |
|  | Per 10 years older | | 1.19 (1.04-1.36) | 0.0137 | 1.16 (1.00-1.34) | 0.0537 | 1.17 (1.02-1.34) | 0.0278 |
| Age group | | |  |  |  |  |  |  |
|  | <40 | | 1.00 | 0.0864 |  |  |  |  |
|  | 40-49 | | 1.28 (0.77-2.12) |  |  |  |  |  |
|  | 50-59 | | 1.54 (0.95-2.49) |  |  |  |  |  |
|  | >=60 | | 1.84 (1.12-3.02) |  |  |  |  |  |
| Year of transplantation (grouped) | | |  |  |  |  |  |  |
|  | 2010-2011 | | 1.00 |  |  |  |  |  |
|  | 2012-2013 | | 1.87 (1.22-2.87) | 0.0041 |  |  |  |  |
|  | 2014-2015 | | 2.97 (1.94-4.55) | <.0001 |  |  |  |  |
|  | 2016-2017 | | 7.55 (4.28-13.34) | <.0001 |  |  |  |  |
| Year of transplantation (per year later) | | | 1.35 (1.24-1.48) | <.0001 | 1.04 (0.95-1.13) | 0.3908 |  |  |
| CCI score | | |  |  |  |  |  |  |
|  | Per point higher | | 1.12 (1.02-1.23) | 0.02 | 0.97 (0.86-1.10) | 0.6679 |  |  |
| Baseline^2^ lymphocytes | | |  |  |  |  |  |  |
|  | Below normal (<1.0*10^9^/L) | | 2.01 (1.40-2.88) | 0.0009 | 1.02 (0.67-1.56) | 0.9146 |  |  |
|  | Normal (1.0-<3.5*10^9^/L) | | 1.00 |  | 1.00 |  |  |  |
|  | Above normal (≥3.5*10^9^/L) | | 1.05 (0.23-4.76) |  | 0.84 (0.18-3.95) | 0.8223 |  |  |
|  | Missing | | 2.29 (1.33-3.95) |  | 0.88 (0.48-1.62) | 0.6811 |  |  |
| Baseline^2^ neutrocytes | | |  |  |  |  |  |  |
|  | Below normal (<1.6*10^9^/L) | | 2.22 (0.73-6.79) | 0.1771 |  |  |  |  |
|  | Normal (1.6-<5.9*10^9^/L) | | 1.00 |  |  |  |  |  |
|  | Above normal (≥5.9*10^9^/L) | | 1.05 (0.73-1.50) |  |  |  |  |  |
|  | Missing | | 1.87 (1.08-3.21) |  |  |  |  |  |
| Baseline^2^ albumin^3^ | | |  |  |  |  |  |  |
|  | Below normal | | 2.81 (1.91-4.12) | <.0001 | 1.33 (0.90-1.97) | 0.1504 |  |  |
|  | Normal | | 1.00 |  | 1.00 |  |  |  |
|  | Above normal | | 0.73 (0.36-1.47) |  | 0.85 (0.43-1.68) | 0.6333 |  |  |
|  | Missing | | 1.29 (0.79-2.12) |  | 1.33 (0.69-2.55) | 0.399 |  |  |
| Pre- transplant CDI | | |  |  |  |  |  |  |
|  | No | | 1.00 | 0.0238 | 1.00 |  | 1.00 |  |
|  | Yes (ever) | | 2.69 (1.20-6.03) |  | 2.37 (1.12-4.99) | 0.0237 | 2.43 (1.17-5.03) | 0.0172 |
|  | Yes, in previous 6 months | | 12.17 (2.88-51.36) |  | 5.75 (1.75-18.89) | 0.0039 | 5.67 (1.89-17.02) | 0.002 |
| Donor-recipient CMV serostatus^4^ | | |  |  |  |  |  |  |
|  | High | | 1.00 | 0.2074 |  |  |  |  |
|  | Intermediate | | 1.25 (0.75-2.08) |  |  |  |  |  |
|  | Low | | 1.23 (0.73-2.06) |  |  |  |  |  |
|  | Missing | | 0.72 (0.35-1.45) |  |  |  |  |  |
| Time-updated | | |  |  |  |  |  |  |
| Time since transplantation | | |  |  |  |  |  |  |
|  | 0-30 days | | 7.00 (4.60-10.67) | <.0001 | 6.60 (4.34-10.03) | <.0001 | 6.64 (4.37-10.10) | <.0001 |
|  | 31-180 days | | 1.00 |  | 1.00 |  | 1.00 |  |
|  | > 180 days | | 0.24 (0.15-0.38) |  | 0.27 (0.17-0.43) | <.0001 | 0.25 (0.16-0.40) | <.0001 |
| CMV | | |  |  |  |  |  |  |
|  | No | | 1.00 | 0.1757 |  |  |  |  |
|  | Yes | | 0.70 (0.42-1.17) |  |  |  |  |  |
|  |  | |  |  |  |  |  |  |
| HSCT | | | | | | | | |
|  | | | Univariate IRR  (95% CI) | P-value | Multivariate IRR  (95% CI) | P-value | Final multivariate model IRR  (95% CI) | P-value |
| Overall | | |  |  |  |  |  |  |
| Transplant type | | |  |  |  |  |  |  |
|  | Non-myeloablative | | 1.00 |  | 1.00 |  | 1.00 |  |
|  | Myeloablative | | 1.50 (0.99-2.27) | 0.06 | 1.71 (1.16-2.53) | 0.0066 | 1.72 (1.17-2.25) | 0.0057 |
| Gender | | |  |  |  |  |  |  |
|  | Male | | 1.00 |  |  |  |  |  |
|  | Female | | 1.05 (0.68-1.60) | 0.82 |  |  |  |  |
| Age at transplant | | |  |  |  |  |  |  |
|  | Per 10 years older | | 0.94 (0.82-1.08) | 0.38 |  |  |  |  |
| Age group | | |  |  |  |  |  |  |
|  | <40 | | 1.00 | 0.3869 |  |  |  |  |
|  | 40-49 | | 1.07 (0.58-1.99) |  |  |  |  |  |
|  | 50-59 | | 1.16 (0.65-2.07) |  |  |  |  |  |
|  | >=60 | | 0.74 (0.42-1.28) |  |  |  |  |  |
| Year of transplantation (grouped) | | |  |  |  |  |  |  |
|  | 2010-2011 | | 1.00 |  | 1.00 |  | 1.00 |  |
|  | 2012-2013 | | 2.59 (1.46-4.60) | 0.0011 | 2.27 (1.31-3.94) | 0.0036 | 2.25 (1.30-3.89) | 0.0036 |
|  | 2014-2015 | | 5.09 (2.89-8.96) | <.0001 | 3.46 (1.51-7.92) | 0.0033 | 3.23 (1.87-5.60) | <.0001 |
|  | 2016-2017 | | 5.26 (2.39-11.59) | <.0001 | 1.83 (0.66-5.08) | 0.2435 | 1.70 (0.77-3.75) | 0.1887 |
| Year of transplantation (per year later) | | | 1.35 (1.24-1.48) | <.0001 |  |  |  |  |
| CCI score | | |  |  |  |  |  |  |
|  | per point higher | | 1.23 (1.04-1.45) | 0.01 | 1.17 (1.02-1.33) | 0.0223 | 1.17 (1.02-1.33) | 0.0224 |
| Baseline^2^ lymphocytes | | |  |  |  |  |  |  |
|  | Below normal (<1.0*10^9^/L) | | 1.11 (0.73-1.69) | 0.76 |  |  |  |  |
|  | Normal (1.0-<3.5*10^9^/L) | | 1.00 |  |  |  |  |  |
|  | Above normal (≥3.5*10^9^/L) | | 0.82 (0.33-2.08) |  |  |  |  |  |
|  | Missing | | Excluded  (5 values only) |  |  |  |  |  |
| Baseline^2^ neutrocytes | | |  |  |  |  |  |  |
|  | Below normal (<1.6*10^9^/L) | | 0.82 (0.52-1.30) | 0.69 |  |  |  |  |
|  | Normal (1.6-<5.9*10^9^/L) | | 1.00 |  |  |  |  |  |
|  | Above normal (≥5.9*10^9^/L) | | 0.89 (0.37-2.15) |  |  |  |  |  |
|  | Missing | | Excluded  (7 values only) |  |  |  |  |  |
| Baseline^2^ albumin^3^ | | |  |  |  |  |  |  |
|  | Below normal | | 1.41 (0.47-4.26) | 0.0009 | 1.13 (0.40-3.21) | 0.8133 |  |  |
|  | Normal | | 1.00 |  | 1.00 |  |  |  |
|  | Above normal | | 0.92 (0.49-1.75) |  | 1.00 (0.55-1.83) | 0.9975 |  |  |
|  | Missing | | 2.67 (1.71-4.15) |  | 0.92 (0.43-1.97) | 0.8393 |  |  |
| Pre- transplant CDI | | |  |  |  |  |  |  |
|  | No | | 1.00 | 0.36 |  |  |  |  |
|  | Yes (ever) | | 0.48 (0.06-4.05) |  |  |  |  |  |
|  | Yes, in previous 6 months | | 1.58 (0.77-3.24) |  |  |  |  |  |
| Donor-recipient CMV serostatus^4^ | | |  |  |  |  |  |  |
|  | High | | 1.00 | 0.35 |  |  |  |  |
|  | Intermediate | | 1.25 (0.75-2.08) |  |  |  |  |  |
|  | Low | | 1.23 (0.73-2.06) |  |  |  |  |  |
|  | Missing | | 0.72 (0.35-1.45) |  |  |  |  |  |
| Time-updated | | |  |  |  |  |  |  |
| Time since transplantation | | |  |  |  |  |  |  |
|  | 0-30 days | | 2.88 (1.84-4.50) | <.0001 | 2.85 (1.83-4.43) | <.0001 | 2.85 (1.83-4.43) | <.0001 |
|  | 31-180 days | | 1.00 |  | 1.00 |  | 1.00 |  |
|  | > 180 days | | 0.21 (0.13-0.33) |  | 0.25 (0.15-0.41) | <.0001 | 0.25 (0.15-0.41) | <.0001 |
| CMV | | |  |  |  |  |  |  |
|  | | No | 1.00 | 0.45 |  |  |  |  |
|  | | Yes | - 1. (0.51-1.36) |  |  |  |  |  |

^1^Due to no CDI cases, pancreas recipients were removed from the analyses.

^2^ Baseline measurements were those taken up to 14 days prior to transplantation.

^3^Levels for albumin: normal: age 18-39: 36-48 g/L, age 40-69: 36-45 g/L, age 70-125: 34-45 g/L, below: levels under normal per age group, above: levels above normal per age group

^4^High for SOT=Donor(D)+/Recipient(R)-, High for HSCT= D-/R+, Intermediate for SOT and HSCT= D+/R+, Low for SOT= D-/R+, Low for HSCT= D+/R-.
