# Supplementary material for: Incidence Rates and Risk Factors of Clostridioides difficile Infection in Solid Organ and Hematopoietic Stem Cell Transplant Recipients
Source: Open Forum Infect Dis. 2019 Feb 19;6(4):ofz086. doi: 10.1093/ofid/ofz086 (PMC6441586; doi:10.1093/ofid/ofz086)
Supplement: Supplementary_Table_3 [file ofz086_suppl_supplementary_table_3.docx]

# Supplementary Table 3

## Nested case-control study: Medication use per transplant type

|  | | | | | SOT | | | | | |
| --- | --- | --- | --- | --- | --- | --- | --- | --- | --- | --- |
|  | | | | | **Lung** | | **Liver** | | **Kidney** | |
|  | | | | | Case  (n=20) | Control  (n=20) | Case  (n=25) | Control  (n=25) | Case  (n=20) | Control  (n=20) |
| Age, median (IQR) | | | | | 48 (45-57) | 55 (43-58) | 51 (46-56) | 49 (46-59) | 51 (36-57) | 56 (44-62) |
| Male, n (%) | | | | | 11 (55) | 11 (55) | 16 (64) | 13 (52) | 13 (65) | 12 (60) |
| Charlson Comorbidity index, median (IQR) | | | | | 1 (1-3) | 1 (1-1) | 3 (3-4) | 4 (3-5) | 2.5 (2.0-3.5) | 3 (2-3) |
| Antibiotics^1^ | | | | |  |  |  |  |  |  |
|  | Received treatment, n (%) | | | | 20 (100) | 20 (100) | 25 (100) | 25 (100) | 20 (100) | 20 (100) |
|  | Median^2^ number of treatment days (IQR) | | | | 39 (26-77) | 38 (20-90) | 16 (9-53) | 20 (9-80) | 17 (6-30) | 8 (4-22) |
| Antibiotic groups^1^: | | | | |  |  |  |  |  |  |
|  | Clindamycin | | | |  |  |  |  |  |  |
|  |  | | | Received treatment, n (%) | 1 (5) | 0 | 0 | 0 | 0 | 0 |
|  |  | | | Median^2^ number of treatment days (IQR) | 16 | - | - | - | - | - |
|  | Fluoroquinolones | | | |  |  |  |  |  |  |
|  |  | | | Received treatment, n (%) | 20 (100) | 18 (90) | 12 (48) | 10 (40) | 6 (30) | 5 (25) |
|  |  | | | Median^2^ number of treatment days (IQR) | 18 (10-29) | 19 (9-24) | 8 (3-37) | 26 (6-54) | 6 (1-10) | 2 (1-3) |
|  | 3^rd^/4^th^ generation cephalosporins | | | |  |  |  |  |  |  |
|  |  | | | Received treatment, n (%) | 3 (15) | 2 (10) | 22 (88) | 24 (96) | 2 (10) | 0 |
|  |  | | | Median^2^ number of treatment days (IQR) | 44 (1-90) | 23 (11-35) | 6 (4-6) | 5 (5-6) | 27 (6-48) | - |
|  | Piperacillin/tazobactam | | | |  |  |  |  |  |  |
|  |  | | | Received treatment, n (%) | 5 (25) | 0 | 3 (12) | 0 | 0 | 0 |
|  |  | | | Median^2^ number of treatment days (IQR) | 20 (6-65) | - | 5 (1-7) | - | - | - |
|  | Carbapenems | | | |  |  |  |  |  |  |
|  |  | | | Received treatment, n (%) | 19 (95) | 19 (95) | 17 (68) | 4 (16) | 4 (20) | 4 (20) |
|  |  | | | Median^2^ number of treatment days (IQR) | 19 (13-26) | 18 (7-24) | 6 (4-11) | 8 (6-12) | 8 (1-14) | 4 (2-7) |
|  | Beta-lactam/Beta-lactamase inhibitor comb. (excl. piperacillin/tazobactam) | | | |  |  |  |  |  |  |
|  |  | | | Received treatment, n (%) | 6 (30) | 6 (30) | 23 (92) | 23 (92) | 8 (40) | 3 (15) |
|  |  | | | Median^2^ number of treatment days (IQR) | 7 (3-9) | 6 (3-9) | 5 (4-6) | 5 (4-6) | 7 (4-10) | 1 (1-7) |
|  | Other antibiotics | | | |  |  |  |  |  |  |
|  |  | | | Received treatment, n (%) | 20 (100) | 20 (100) | 20 (80) | 17 (68) | 19 (95) | 20 (100) |
|  |  | | | Median^2^ number of treatment days (IQR) | 33 (21-59) | 35 (19-58) | 11 (5-26) | 57 (7-80) | 17 (5-24) | 9 (4-22) |
| Number of different antibiotic medications prescribed^3^ (IQR) | | | | | 4 (3-4) | 3 (3-4) | 4 (3-5) | 3 (2-4) | 2 (1-3) | 1 (1-2) |
|  | 1-2 (%) | | | | 0 (0) | 2 (10) | 3 (12) | 7 (28) | 15 (75) | 16 (80) |
|  | 3-4 (%) | | | | 16 (80) | 18 (90) | 14 (56) | 15 (60) | 5 (25) | 4 (20) |
|  | >5 (%) | | | | 4 (20) | 0 (0) | 8 (32) | 3 (12) | 0 | 0 |
| Proton pump inhibitors^1^ | | | | |  |  |  |  |  |  |
|  | Received treatment, n (%) | | | | 20 (100) | 20 (100) | 25 (100) | 24 (96) | 20 (100) | 20 (100) |
|  | Median^2^ number of treatment days (IQR) | | | | 33 (13) | 35 (9-52) | 89 (25-90) | 64 (13-90) | 69 (10-90) | 16 (6-64) |
| Steroids^1^ | | | | |  |  |  |  |  |  |
|  | Received treatment, n (%) | | | | 20 (100) | 20 (100) | 25 (100) | 25 (100) | 20 (100) | 20 (100) |
|  | Median^2^ number of treatment days (IQR) | | | | 48 (33-90) | 36 (30-51) | 15 (12-35) | 5 (12-25) | 18 (8-23) | 10 (6-25) |
| Antimycotics^1^ | | | | |  |  |  |  |  |  |
|  | Received treatment, n (%) | | | | 20 (100) | 19 (95) | 24 (96) | 24 (96) | 4 (20) | 2 (10) |
|  | Median^2^ number of treatment days (IQR) | | | | 31 (11-48) | 19 (7-35) | 10 (8-15) | 8 (7-11) | 6 (3-9) | 9 (5-13) |
| Parenteral nutrition^1^ | | | | |  |  |  |  |  |  |
|  | Received treatment, n (%) | | | | 0 | 0 | 1 (4) | 0 | 0 | 0 |
|  | Median^2^ number of treatment days (IQR) | | | | - | - | 5 (-) | - | - | - |
| Received laxatives^4^ | | | | | 14 (70) | 13 (65) | 10 (40) | 7 (28) | 14 (70) | 14 (70) |
|  | | | | | **HSCTCT** | | | | | |
|  | | | | | **Myeloablative** | | **Non-Myeloablative** | |  | |
|  | | | | | Case  (n=25) | Control  (n=25) | Case  (n=17) | Control  (n=17) |  |  |
| Age, median (IQR) | | | | | 43 (32-47) | 43 (34-51) | 60 (55-66) | 62 (57-66) |  |  |
| Male, n (%) | | | | | 14 (56) | 14 (56) | 10 (59) | 8 (47) |  |  |
| Charlson Comorbidity index, median (IQR) | | | | | 2 (2-3) | 2 (2-2) | 2 (2-2) | 2 (2-3) |  |  |
| Antibiotics^1^ | | | | |  |  |  |  |  |  |
|  | | Received treatment, n (%) | | | 25 (100) | 25 (100) | 17 (100) | 17 (100) |  |  |
|  | | Median^2^ number of treatment days (IQR) | | | 59 (50-75) | 53 (40-65) | 59 (36-84) | 48 (25-65) |  |  |
| Antibiotic groups^1^ | | | | |  |  |  |  |  |  |
|  | | Clindamycin | | |  |  |  |  |  |  |
|  | |  | Received treatment, n (%) | | 1 (4) | 2 (8) | 0 | 2 (12) |  |  |
|  | |  | Median^2^ number of treatment days (IQR) | | 9 (-) | 7 (4-9) | - | 23 (17-29) |  |  |
|  | | Fluoroquinolones | | |  |  |  |  |  |  |
|  | |  | Received treatment, n (%) | | 21 (84) | 20 (80) | 16 (94) | 17 (100) |  |  |
|  | |  | Median^2^ number of treatment days (IQR) | | 23 (14-39) | 18 (10-37) | 37 (16-52) | 27 (16-38) |  |  |
|  | | 3^rd^/4^th^ generation cephalosporins | | |  |  |  |  |  |  |
|  | |  | Received treatment, n (%) | | 24 (96) | 16 (64) | 5 (29) | 2 (12) |  |  |
|  | |  | Median^2^ number of treatment days (IQR) | | 7 (5-11) | 8 (5-14) | 11 (9-11) | 5 (3-6) |  |  |
|  | | Piperacillin/tazobactam | | |  |  |  |  |  |  |
|  | |  | Received treatment, n (%) | | 7 (28) | 9 (36) | 8 (47) | 7 (41) |  |  |
|  | |  | Median^2^ number of treatment days (IQR) | | 1 (1-5) | 8 (3-14) | 4 (2-6) | 4 (4-8) |  |  |
|  | | Carbapenems | | |  |  |  |  |  |  |
|  | |  | Received treatment, n (%) | | 23 (92) | 20 (80) | 8 (47) | 8 (47) |  |  |
|  | |  | Median^2^ number of treatment days (IQR) | | 13 (8-20) | 16 (8-21) | 10 (5-17) | 8 (4-11) |  |  |
|  | | Beta-lactam/Beta-lactamase inhibitor comb. (excl. piperacillin/tazobactam) | | |  |  |  |  |  |  |
|  | |  | Received treatment, n (%) | | 20 (80) | 19 (76) | 10 (59) | 12 (71) |  |  |
|  | |  | Median^2^ number of treatment days (IQR) | | 15 (8-20) | 14 (7-18) | 21 (7-28) | 12 (6-27) |  |  |
|  | | Other antibiotics | | |  |  |  |  |  |  |
|  | |  | Received treatment, n (%) | | 25 (100) | 25 (100) | 17 (100) | 17 (100) |  |  |
|  | |  | Median^2^ number of treatment days (IQR) | | 29 (20-60) | 28 (16-40) | 31 (19-56) | 10 (7-39) |  |  |
| Number of different antibiotic medications prescribed^3^ (IQR) | | | | | 5 (4-6) | 5 (4-5) | 4 (3-5) | 4 (3-5) |  |  |
|  | | 1-2 (%) | | | 0 (0) | 2 (8) | 4 (24) | 1 (6) |  |  |
|  | | 3-4 (%) | | | 7 (28) | 8 (32) | 7 (40) | 10 (59) |  |  |
|  | | >5 (%) | | | 18 (72) | 15 (60) | 6 (35) | 6 (35) |  |  |
| Proton pump inhibitors^1^ | | | | |  |  |  |  |  |  |
|  | | Received treatment, n (%) | | | 22 (88) | 23 (92) |  |  |  |  |
|  | | Median^2^ number of treatment days (IQR) | | | 47 (31-89) | 35 (22-70) |  |  |  |  |
| Steroids^1^ | | | | |  |  |  |  |  |  |
|  | | Received treatment, n (%) | | | 17 (68) | 10 (40) | 7 (41) | 7 (41) |  |  |
|  | | Median^2^ number of treatment days (IQR) | | | 8 (2-20) | 14 (6-21) | 10 (6-31) | 8 (3-33) |  |  |
| Antimycotics^1^ | | | | |  |  |  |  |  |  |
|  | | Received treatment, n (%) | | | 25 (100) | 25 (100) | 17 (100) | 16 (94) |  |  |
|  | | Median^2^ number of treatment days (IQR) | | | 51 (36-66) | 49 (34-69) | 61 (36-89) | 38 (16-60) |  |  |
| Parenteral nutrition^1^ | | | | |  |  |  |  |  |  |
|  | | Received treatment, n (%) | | | 20 (80) | 22 (88) | 3 (18) | 1 (6) |  |  |
|  | | Median^2^ number of treatment days (IQR) | | | 14 (8-24) | 13 (6-20) | 9 (8-24) | 6 (-) |  |  |
| Received laxatives^4^ | | | | | 7 (28) | 5 (20) | 8 (47) | 2 (12) |  |  |

^1^Within 90 days prior to CDI for cases or corresponding time-period relative to transplantation for controls.

^2^The median in this table is based only on those receiving the medication in question. Different types of medications within medication groups are not counted cumulatively; max median number of days is 90.

^3^Number of different antibiotic medications prescribed within 90 days prior to CDI for cases or corresponding time-period relative to transplantation for controls.

^4^If patients received laxatives up to 7 days prior to CDI for cases or corresponding time-period relative to transplantation for controls.
