# Supplementary material for: Incidence Rates and Risk Factors of Clostridioides difficile Infection in Solid Organ and Hematopoietic Stem Cell Transplant Recipients
Source: Open Forum Infect Dis. 2019 Feb 19;6(4):ofz086. doi: 10.1093/ofid/ofz086 (PMC6441586; doi:10.1093/ofid/ofz086)
Supplement: Supplementary_Table_4 [file ofz086_suppl_supplementary_table_4.docx]

# Supplementary Table 4

# Results of the univariate and multivariate logistic regression analyses for the nested case-control study of medication-use 90 days prior to CDI

| SOT^1^ | | | | |
| --- | --- | --- | --- | --- |
|  | Univariate OR (95% CI) | P-value | Multivariate^2^ OR (95% CI) | P-value |
| Total number of antibiotic days^3^ | 1.00 (0.98-1.01) | 0.5434 |  |  |
| Total number of days with^3, 4^: |  |  |  |  |
| Clindamycin | . | . |  |  |
| Fluoroquinolones | 1.01 (0.99-1.03) | 0.5423 |  |  |
| 3rd/4th generation cephalosporins | 1.03 (0.98-1.07) | 0.2780 |  |  |
| Piperacillin/tazobactam | . | . |  |  |
| Carbapenems | 1.09 (1.02-1.18) | 0.0184 | 1.04 (0.98-1.09) | 0.1923 |
| Beta-lactam/Beta-lactamase inhibitor comb. (excl. piperacillin/ tazobactam) | 1.02 (0.97-1.07) | 0.4188 |  |  |
| Other antibiotics | 0.99 (0.98-1.01) | 0.2865 |  |  |
| Number of different antibiotic medications prescribed^5^ | 1.88 (1.23-2.86) | 0.0036 | 1.66 (1.07-2.57) | 0.0242 |
| Total number of days with antimycotics^3^ | 1.02 (0.99-1.05) | 0.1252 |  |  |
| Total number of days with steroid treatment^3^ | 1.01 (1.00-1.03) | 0.1136 |  |  |
| Total number of days with PPI’s^3^ | 1.01 (1.00-1.02) | 0.0623 | 1.01 (1.00-1.03) | 0.0202 |
| Total number of days with parenteral nutrition^3^ | . | . |  |  |
| Laxatives^6^ | 1.50 (0.53-4.21) | 0.4417 |  |  |
| HSCT | | | | |
|  | Univariate OR (95% CI) | P-value | Multivariate^2^ OR (95% CI) | P-value |
| Total number of antibiotic days^3^ | 1.03 (1.00-1.06) | 0.0534 |  |  |
| Total number of days with^3,4^: |  |  |  |  |
| Clindamycin | 0.89 (0.72-1.09) | 0.2604 |  |  |
| Fluoroquinolones | 1.02 (0.99-1.05) | 0.197 |  |  |
| 3rd/4th generation cephalosporins | 1.07 (0.98-1.18) | 0.1407 |  |  |
| Piperacillin/tazobactam | 0.89 (0.77-1.03) | 0.1311 |  |  |
| Carbapenems | 1.02 (0.98-1.07) | 0.363 |  |  |
| Beta-lactam/Beta-lactamase inhibitor comb. (excl. piperacillin/ tazobactam) | 1.01 (0.98-1.04) | 0.6063 |  |  |
| Other antibiotics | 1.03 (1.00-1.06) | 0.04 | 1.02 (1.00-1.05) | 0.0491 |
| Number of different antibiotic medications prescribed^5^ | 1.14 (0.81-1.61) | 0.4451 |  |  |
| Total number of days with antimycotics^3^ | 1.01 (0.99-1.04) | 0.1769 |  |  |
| Total number of days with steroid treatment^3^ | 1.01 (0.98-1.03) | 0.7071 |  |  |
| Total number of days with PPI’s^3^ | 1.02 (1.00-1.03) | 0.0725 | 1.01 (1.00-1.03) | 0.1701 |
| Total number of days with parenteral nutrition^3^ | 1.03 (0.96-1.10) | 0.44 |  |  |
| Laxatives^6^ | 2.60 (0.93-7.293) | 0.0694 | 2.91 (0.94-9.00) | 0.0645 |

^1^ Due to no CDI cases there were no pancreas recipients in the nested case-control study.

^2^Also adjusted for age, sex, transplant/conditioning type, year of transplantation and the Charlson Comorbidity Index

^3^Within 90 days prior to CDI for cases or corresponding time-period relative to transplantation for controls. Different types of medications within medication groups are not counted cumulatively; maximum number of days is 90. Within the logistic regression ORs are per extra day of treatment.

^4^The antibiotics included in the different subgroups are listed in Supplementary Material 2.

^5^Number of different antibiotic medications prescribed within 90 days prior to CDI for cases or corresponding time-period relative to transplantation for controls. Within the logistic regression ORs are per additional antibiotic.

^6^If patients received laxatives up to 7 days prior to CDI for cases or corresponding time-period relative to transplantation for controls.
