# Supplementary material for: Incidence Rates and Risk Factors of Clostridioides difficile Infection in Solid Organ and Hematopoietic Stem Cell Transplant Recipients
Source: Open Forum Infect Dis. 2019 Feb 19;6(4):ofz086. doi: 10.1093/ofid/ofz086 (PMC6441586; doi:10.1093/ofid/ofz086)
Supplement: Supplementary_Material_1 [file ofz086_suppl_supplementary_material_1.docx]

# Supplementary Material 1

## Data Sources

The PERSIMUNE data warehouse collects data generated from routine patient treatment available for data extraction, as well as additional data from national registries and clinical databases. Patients were linked across data sources using their unique ten-digit civil registration number given to all Danish residents before pseudonymization.

This project is based on data from the following data sources:

**National data**

- MedCom (<http://medcom.dk/medcom-in-english>): established in 1994, links and provides data from multiple electronic data sources (authorities, organizations and private firms) associated with the Danish healthcare sector. MedCom delivers the following data:
  - Biochemistry from all Danish Regions and private actors:
    - Capital Region of Denmark, complete since September 2014
    - Capital Region of Denmark’s Elektive Laboratorium, complete since 2010
    - Region Zealand, complete since February 2014
    - North Denmark Region, complete since December 2014
    - Region of Southern Denmark, complete since 2013
    - Central Denmark Region, complete since 2009
  - Microbiology
    - Danish Microbiology Database (MiBa)[1], has gathered all data on microbiological samples in Denmark and is complete for all hospital microbiology departments since 2010 (however Statens Serum Institut was first included from 2013 onwards)
- National Patient Registry (LPR)[2]: established in 1977 and contains data on administrative data and admissions, diagnoses and procedures for all patients treated in the Danish healthcare system.
- Civil Registration System (CRS)[3]: established in 1968 and contains data on death, emigration and loss-to-follow-up (i.e. disappearance according to the Danish authorities) for all Danish residents.

**Regional data**

- Labka: contains biochemistry data from the Capital Region of Denmark and is complete from 2008 onwards. Labka was used in addition to the biochemistry data delivered from MedCom (from the Capital Region of Denmark, see National data above) and was also used to cross-reference the data delivered from MedCom (from the Capital Region of Denmark, see National data above).
- Sundhedsdatabanken: contains administrative data on admissions, outpatient visits, procedures and other administrative data from the Capital Region of Denmark. Sundhedsdatabanken was introduced in 2008.

**Local data**

- Management of post-Transplant infections in Collaborating Hospitals (MATCH): established in 2010. Includes all solid organ and allogeneic haematopoietic stem cell transplantations conducted at Rigshospitalet, Copenhagen University Hospital. Contains data on transplantation date, transplantation type, date of birth and biochemistry results for viral infections.

**Manual collection of medication data:**

- EPM1 and EPM3: both are clinical electronic medication systems and databases for hospitals within the Capital Region of Denmark. Intensive care departments have not used these medication systems but have written reports in the patient notes. EMP1 was established in 2008 and was replaced by EMP3 in 2011 which in turn was replaced by SP(EPIC) during the year of 2016- 2017.
- OPUS: a clinical electronic patient notes/records system and database for hospitals within the Capital Region of Denmark. Established in 2009 and replaced by SP(EPIC) during the year of 2016- 2017.
- SP(EPIC): a clinical electronic patient record system, combines both medication and clinical notes in one system and database. Rolled out at hospitals within the Capital Region of Denmark during the year of 2016-2017.
- E-journalen: an electronic patient records system consisting of copies of patient notes/records from all hospitals in Denmark.

## References

1. Voldstedlund M, Haarh M, Molbak K. The Danish Microbiology Database (MiBa) 2010 to 2013. Euro surveillance : bulletin Europeen sur les maladies transmissibles = European communicable disease bulletin **2014**; 19(1).

2. Lynge E, Sandegaard JL, Rebolj M. The Danish National Patient Register. Scandinavian journal of public health **2011**; 39(7 Suppl): 30-3.

3. Pedersen CB. The Danish Civil Registration System. Scandinavian journal of public health **2011**; 39(7 Suppl): 22-5.
