# Supplementary material for: Incidence Rates and Risk Factors of Clostridioides difficile Infection in Solid Organ and Hematopoietic Stem Cell Transplant Recipients
Source: Open Forum Infect Dis. 2019 Feb 19;6(4):ofz086. doi: 10.1093/ofid/ofz086 (PMC6441586; doi:10.1093/ofid/ofz086)
Supplement: Supplementary_Material_2 [file ofz086_suppl_supplementary_material_2.docx]

# Supplementary Material 2

## Additional information regarding medical data collection

A total of 25 cases were included per transplant type. For transplant types with more than 25 cases, those with the most recent transplant were included. When medication had a missing stop date the average prescribed treatment time for that particular medication was used. Medications prescribed for as needed use (“Pro re nata”, PRN) were counted as regular prescriptions. Antibiotics were grouped into the following subcategories: clindamycin, fluoroquinolones, 3^rd^/4^th^ generation cephalosporins, piperacillin/tazobactam, carbapenems, beta-lactam/beta-lactamase inhibitor combinations (minus piperacillin/tazobactam) and others. The total number of days treated with each subcategory was calculated, as well as the number of different antibiotic types given within the 90-day period.

Fluoroquinolones could include: Ofloxacin, Ciprofloxacin, Levofloxacin and Moxifloxacin. 3^rd^/4^th^ generation cephalosporins could include: Cefotaxime, Ceftazidime, Ceftriaxone, Ceftizoxime and Cefepime. Carbapenems could include: Meropenem, Ertapenem, Doripenem and Imipenem with enzyme inhibitor. Beta-lactam/beta-lactamase inhibitor combinations (minus piperacillin/tazobactam) could include: Ampicillin, Pivampicillin, Amoxicillin, Pivmecillinam, Mecillinam, Temocillin, Benzylpenicillin, Phenoxymethylpenicillin, Benzathin benzylpenicillin, Dicloxacillin, Cloxacillin, Flucloxacillin, Ampicillin and enzyme inhibitor, Amoxicillin with enzyme inhibitor. Other antibiotics could include: Azithromycin, Aztreonam, Cefalexin, Cefuroxime, Chloramphenicol, Clarithromycin, Colistin, Dapsone, Daptomycin, Doxycycline, Erythromycin, Fidaxomicin, Fusidic acid, Gentamicin, Isoniazid, Linezolid, Methronidazole, Metronidazole, Nitrofurantoin, Rifampicin, Rifaximin, Roxithromycin, Sulfamethizole, Sulfamethoxazole and trimethoprim, Tigecycline, Tobramycin and Vancomycin.

## Standard immunosuppression regimes

***Liver transplantation***

Following liver transplantation, a single dose of methylprednisolone 1,000 mg is given intraoperatively. In the days following the transplantation, prednisolone is tapered gradually from 200 mg on day 1 to 30 mg on day 5. For the remaining first month, 20 mg is given daily tapered to 15, 10 and 7.5 mg daily until month 6. For the next 6 months, 5 mg is given daily after which the drug is discontinued. Tacrolimus is given twice daily, aiming at through levels of 10–12 ng/mL in the first month, 8–10 ng/mL in month 2, 7–9 ng/mL in months 3–6, 6–8 ng/mL in months 7–12 and 4–6 ng/mL after 1 year. Mycophenolate mofetil is given twice daily at a dosage of 1,000 mg continuously.

***Heart transplantation***

Following heart transplantation, thymoglobulin 1.5 mg/kg for 3 days with 1 g methylprednisolone for 2 days followed by 125 mg for an additional 3 days is used. Prednisone 0.2 mg/kg, tapered to 0.1 mg/kg after 3 months and to zero after 1 year, and mycophenolate mofetil 1–1.5 g × 2 daily continuously. Cyclosporine (or tacrolimus) is used adjusted to through blood levels between 200 and 350 ng/mL (10-15 ng/mL) for the first 6 weeks then reduced to 150–250 ng/mL (8-12 ng/mL) and again to 100 ng/mL (5-8 ng/mL) after one year.

***Lung transplantation***

Following lung transplantation, thymoglobulin 1.5 mg/kg for 3 days with 500 mg methylprednisolone for 1 day followed by 125 mg methylprednisolone each day for 3 days is used. Prednisone 15 mg daily tapered to 5 mg daily over 4 weeks. Azathioprine 1.5 mg/kg from postoperative day 1. Cyclosporine (or tacrolimus) from postoperative day 1 with target range 200–240 ng/mL (10-15 ng/mL) for first 3 months, 150–200 ng/ml (8-12 ng/mL) for months 4–12, and again to 100-150 ng/mL (5-8 ng/mL) after one year.

***Kidney transplantation***

Following kidney transplantation typically, induction was given with either five doses of daclizumab or two doses of basiliximab and methylprednisolone 250-500 mg immediately before transplantation. Maintenance immunosuppression was started at day 1 as triple therapy with cyclosporine or tacrolimus, mycophenolate mofetil and steroids.

***HSCT***

Following myeloablative HSCT, cyclosporine adjusted to through blood level between 200 and 400 ng/mL is standard of care. A short-course of methotrexate is given post-transplant: 15 mg/m^2^ day 1, 10 mg/m^2^ day 3, 6 and 11. After non-myeloablative HSCT, either cyclosporine or tacrolimus is adjusted to 400 ng/mL and 10-14 ng/mL, respectively. Mycophenolate mofetil 1-1.5 mg twice a day (related donor) or three times a day (unrelated donor) is given until day 28, and then stopped (related donor) or tapered to day 98 (unrelated donor).

Prednisone is added in case of graft-versus-host disease (GVHD); starting at 2 mg/kg for acute GVHD, and 1 mg/kg for chronic GVHD. After clinical response, prednisone is tapered. If GVHD is steroid-refractory, infliximab 10 mg/kg/week is added.
