# Supplementary material for: Incidence Rates and Risk Factors of Clostridioides difficile Infection in Solid Organ and Hematopoietic Stem Cell Transplant Recipients
Source: Open Forum Infect Dis. 2019 Feb 19;6(4):ofz086. doi: 10.1093/ofid/ofz086 (PMC6441586; doi:10.1093/ofid/ofz086)
Supplement: Supplementary_Material_3 [file ofz086_suppl_supplementary_material_3.docx]

# Supplementary Material 3

## Additional information regarding CDI testing

At the start of the follow-up period (2010-2011), the majority of microbiology departments cultured stool samples and if *C. difficile* was found, toxin gene testing was performed. After 2012 there was an increase in testing directly for *C. difficile* genes via PCR, without performing a culture, as shown in the graph below.

Percentage of the different testing methods used for all positive cases per study year (*2017 includes only January and until February 21^st^, <4 CDI cases) .

The number of actual cases are shown in the sub-bars.

Toxin-only= a positive PCR test for C. difficile toxin genes without a culture of C. difficile.

Toxigenic culture= a culture with growth of C. difficile and which tested positive for C. difficile toxin genes.

Culture-only= Cultures with growth of *C. difficile* and no performed toxin test.
